# Association of inflammatory markers with all-cause mortality and cardiovascular mortality in postmenopausal women with osteoporosis or osteopenia

**DOI:** 10.1186/s12905-023-02631-6

**Published:** 2023-09-14

**Authors:** Li Qu, Xiaole Zuo, Jing Yu, Ran Duan, Botao Zhao

**Affiliations:** 1 Department of Laboratory, Taian Maternity And Child Health Hospital, No.386 Longtan Road, Gaoxin District, Tai’an, 271000 People’s Republic of China; 2 Department of Blood Transfusion, Taian Maternity And Child Health Hospital, Tai’an, 271000 People’s Republic of China; 3 Department of Newborn Disease Screening, Taian Maternity And Child Health Hospital, Tai’an, 271000 People’s Republic of China

**Keywords:** Inflammatory markers, All-cause mortality, Cardiovascular mortality, Postmenopausal osteoporosis

## Abstract

**Background:**

The objective of the present study was to investigate whether associations exist between inflammatory biomarkers and all-cause mortality and cardiovascular disease (CVD) mortality in women with postmenopausal osteoporosis (PMOP) or osteopenia.

**Methods:**

In this retrospective cohort study, data were obtained from the National Health and Nutrition Examination Survey database from the years 2007 to 2010, 2013 to 2014, and 2017 to 2018. The inflammatory biomarkers including neutrophil/lymphocyte ratio (NLR), platelet/lymphocyte ratio (PLR), monocyte/lymphocyte ratio (MLR), neutrophil × platelet/lymphocyte (SII), neutrophil × monocyte/lymphocyte (SIRI), and neutrophil × monocyte × platelet/lymphocyte ratio (AISI) were calculated.

**Results:**

A total of 2,834 women were included, with a median survival of 113.51 (3.15) months. During follow-up, 602 women died of all-cause mortality and 185 women died of CVD. NLR, MLR, SIRI, and AISI were significantly associated with all-cause mortality in postmenopausal women with osteoporosis or osteopenia. NLR, MLR, SIRI, and AISI were related to CVD mortality in postmenopausal women with osteoporosis or osteopenia (All *P* < 0.05). Based on the results of the subgroup analysis, AISI, SIRI, and MLR were associated with all-cause mortality and CVD mortality in postmenopausal women with PMOP or osteopenia who had a history of CVD and diabetes. AISI, SII, MLR, and NLR were associated with all-cause mortality and CVD mortality in PMOP or osteopenia women with a body mass index (BMI) > 25 kg/m^2^. PLR was associated with all-cause mortality in PMOP or osteopenia women aged ≥ 65 years.

**Conclusion:**

Inflammatory biomarkers were correlated with mortality risk in the PMOP or osteopenia population. This finding may be helpful for the prognosis management of PMOP or osteopenia in postmenopausal women.

**Supplementary Information:**

The online version contains supplementary material available at 10.1186/s12905-023-02631-6.

## Background

Postmenopausal osteoporosis (PMOP) is a common debilitating disease characterized by low bone mineral density (BMD) and destruction of bone structure caused by osteopenia due to age-related decline in female ovarian function [1]. With an aging population, the incidence of osteoporosis is increasing every year and represents a heavy economic burden worldwide [2, 3]. Epidemiological studies have shown that approximately 50% of postmenopausal women over the age of 50 are affected by osteoporosis [4, 5]. PMOP increases the risk of fracture in postmenopausal women. PMOP increases the risk of fracture in postmenopausal women. In addition, women with PMOP have increased mortality, including cardiovascular disease (CVD) mortality [6, 7]. Therefore, the evaluation of factors associated with mortality in women with PMOP is of great clinical and public health importance.

With the sudden drop in estrogen secretion after menopause [8, 9], women’s bodies are in a state of chronic low-grade inflammation [10]. Bone immunology indicates that inflammatory mediators play an important role in osteoporosis [11]. Recently, several available inflammatory biomarkers, derived from routine blood counts, have been found to be associated with osteoporosis or fractures [1, 12–16]. Neutrophil/lymphocyte ratio (NLR), platelet/lymphocyte ratio (PLR), monocyte/lymphocyte ratio (MLR), systemic immune-inflammation index (SII), systemic inflammation response index (SIRI), and neutrophil × monocyte × platelet/lymphocyte ratio (AISI) are blood-cell indexes derived from complete blood count (CBC) that may better express the inflammatory state of a disease than the counting of a single cell [17]. Accumulating studies have also revealed the association between inflammatory biomarkers and outcomes in women with fractures. A study reported that a high PLR was associated with an increased risk of 1-year all-cause mortality in older adults with a hip fracture [18]. Higher pre- and post-operative NLRs correlated with higher risk of long-term mortality after hip fracture surgery in the geriatric population [19]. Increased SII is associated with increased all-cause mortality in older adults after hip fracture surgery [20]. In the geriatric hip fracture population, admission MLRs were significantly higher in the 30-day and 1-year mortality groups, according to Bingol et al. [21]. The relationship between inflammatory biomarkers and mortality in women with PMOP or osteoporosis deserves further study. In addition, NLR and PLR may have a high value in predicting the prognosis of CVDs [22]. A study by Oylumlu et al. reported that PLR may be useful in long-term risk classification of patients presenting with acute coronary syndrome [23]. An analysis from the database indicated that elevated SII of patients with acute ischemic stroke increased the risk of 30-day mortality [24]. However, few studies have investigated the association between inflammatory biomarkers and CVD mortality in women with PMOP or osteopenia. Research into the factors associated with mortality in women with PMOP or osteopenia may be helpful in the early assessment of the prognosis of women with PMOP.

Herein, the purpose of the current study was to investigate whether association exists between inflammatory biomarkers and all-cause mortality and CVD mortality in women with PMOP or osteopenia.

## Methods

### Study design and participants

This was a retrospective cohort study. Data were extracted from the National Health and Nutrition Examination Survey (NHANES), which aims to evaluate the nutrition and health status of general United States residents. The surveys were approved by the National Center for Health Statistics Research. Details of study implementation are available for online access to NHANES Questionnaires, Datasets, and Related Documentation (cdc.gov). We extracted data from the NHANES from 2007 to 2010, 2013 to 2014, and 2017 to 2018. Inclusion criteria were: (I) age ≥ 45 years old; (2) postmenopausal women; (3) osteoporosis or osteopenia; (4) data with information on the neutrophil count, platelet count, lymphocyte count, and monocyte count. Exclusion criteria were: (1) missing information on survival; (2) missing information on important covariates. Since all data are downloaded from the public database, this research does not need the approval of the ethics committee from our hospital.

### Definitions and measurements

#### BMD testing

Total BMD testing was performed by dual-energy X-ray absorptiometry (DEXA) using Hologic QDR 4500A bone densitometers and Apex software (version: 3.2) throughout by certified radiology technologists. A high level of quality control was maintained throughout the DXA data collection and scan analysis, including a rigorous phantom scanning schedule. Additional information is available on the NHANES website [25]. The T-scores for osteoporosis were also calculated by standardizing the mean and standard deviation [mean (SD)] of the femoral neck and femur in women in reference [26], femur female mean (SD) = 0.94 (0.122), femur neck female mean (SD) = 0.86 (0.12)]. Lumbar BMD was standardized using the mean and standard deviation [mean (SD)] of a previous reference [27] [mean (SD) = 1.065 (0.122)].

#### Osteopenia and osteoporosis

Osteoporosis was referred to a T-score ≤ -2.5, and osteopenia was defined as a T-score < -2.5 < -1.0.

#### Measurements of inflammatory indices

NLR was the ratio of neutrophil count to lymphocyte count. PLR was the ratio of platelet count to lymphocyte count. MLR was the ratio of monocyte count to lymphocyte count. SII was calculated by multiplying the neutrophil and platelet counts and dividing the result by the lymphocyte count. SIRI was calculated by multiplying the neutrophil count by the monocyte count and dividing the result by the lymphocyte count. The AISI was calculated by multiplying the neutrophil, monocyte, and platelet counts and dividing the result by the lymphocyte count.

### Potential covariates

A number of variables were extracted from the database, including (1) baseline characteristics: age, race, educational level, marital status, family income, smoking status (yes or no), body mass index (BMI) categories, metabolic equivalent (MET, met*min), central obesity (yes or no), history of diabetes (yes or no), history of CVD (yes or no), family history of CVD (yes or no), parents ever had a fracture (yes or no), energy (kcal), occupation, antihypertensive drug (yes or no), lipid-lowering drug (yes or no), osteoporosis drug (yes or no), and severity of osteoporosis (osteoporosis and osteopenia); (2) laboratory parameters: high-density lipoprotein cholesterol (HDL-C, mmol/L), total cholesterol, serum glucose, systolic blood pressure (SBP, mmHg), and diastolic blood pressure (DBP, mmHg).

Age was given in years. Due to the small sample size in the groups which included Other Hispanic and Other Race: Including Multi-Racial, these two categories were combined. Thus, in this study, the race was grouped into 4 categories: Mexican American, non-Hispanic White, non-Hispanic Black, and other races including multi-racial. Education was grouped into 5 categories: less than 9th grade, 9th-11th grade, high school grade/general equivalent diploma or equivalent, some college or associate of arts degree, and college graduate or above. Marital status was grouped into 3 categories: married, never married, and others: (widowed, divorced, separated, living with a partner). Family income was determined according to the family poverty income ratio (PIR), which examines family income in relation to family size [28]. Smokers were defined as having smoked more than 100 cigarettes in their lifetime. and smoking some days or every day, while nonsmokers smoked less than 100 cigarettes in their lifetime. BMI was calculated using the equation BMI = weight (kg) / height (m)^2^ [29]. Underweight was defined as BMI < 18.5, normal weight was as 18.5 ≤ BMI < 25, overweight was as 25 ≤ BMI < 30, and obesity was as BMI ≥ 30 kg/m^2^. The criteria for central obesity were waist circumference ≥ 88 cm for women and ≥ 102 cm for men [30]. Participants’ levels of total physical activity were evaluated by determining the MET hours/day (METs score), which was calculated by multiplying the time spent on activities (/day) by MET intensity. Occupation included an employee of the private company, working for the government, self-employed or family business/farm, and unknown. History of diabetes was defined as answering yes to the DIQ010. CVD diagnoses were considered for participants with one of the following conditions including congestive heart failure, coronary heart disease, angina/angina pectoris, heart attack, and stroke [31]. Total cholesterol as the exposure variable was measured via a serum sample by Roche Cobas 6000, which is an enzymatic method where esterified cholesterol is converted to cholesterol by cholesterol esterase and then acted upon by cholesterol oxidase to produce cholest-4-en-3-one and hydrogen peroxide [32]. HDL-C levels were determined using polyethylene glycol-coupled cholesteryl esterase, cholesterol oxidase, and sulfated alpha-cyclodextrin [33]. During the NHANES health examination, blood samples were obtained by venipuncture and immediately centrifuged, ali-quoted, and frozen to -20 C. The frozen serum and plasma samples were then shipped on dry ice to central laboratories and stored at -70 C until analysis. Samples prior to 2007 were analyzed by the Johns Hopkins University laboratory, and from 2007, by the University of Minnesota laboratory. Detailed processing steps can be found in the description of plasma sample components on the NHANES official website (https://www.cdc.gov/nchs/nhanes/about_nhanes.htm). Meanwhile, the NHANES project team employs several different approaches to test the quality of assays performed by the laboratory, including but not limited to conducting a second examination of previously examined participants.

### Laboratory quality control

NHANES quality control and quality assurance protocols (QA/QC) meet the requirements of the Clinical Laboratory Improvement Act 1988. Detailed QA/QC instructions are discussed in the NHANES LPM (NHANES Laboratory Procedures Manual (https://www.cdc.gov/nchs/nhanes/index.htm).

### Outcome ascertainment and follow-up

The outcomes in this study are all-cause mortality and CVD mortality. All-cause mortality was defined as death from any cause during follow-up. CVD mortality was defined as death in which CVD was listed as the underlying cause of death on the death certificate. The status of death and the time of follow-up were extracted through the public-use linked mortality file obtained from the NCHS (https://www.cdc.gov/nchs/data-linkage/mortality.htm#print) and matched with the ID of participants from the NHANES database. The time of mortality was determined as the interval between the date of the interview and the date of death or the last follow-up date. The median survival was 113.51 (3.15) months.

### Statistical analysis

For baseline data, continuous variables were expressed as the mean and standard error (SE), and categorical variables were expressed as the number of cases and constituent ratio [N (%)]. A weighted Chi-square test (for categorical variables) or the weighted T-test (for continuous variables) were used to examine the differences between different groups. Statistical analyses were performed using SAS 9.4 version (SAS Institute Inc., Cary, NC, USA) and R version 4.2.0 (2022–04-22 ucrt). A two-tailed *P* < 0.05 was considered statistically significant.

The weighted Cox regression analysis was used to analyze the hazard ratio (HR) and 95% confidence interval (CI). A significant variable by univariate analysis was further analyzed by multivariate analysis to explore the association between NLR, PLR, MLR, SII, SIRI, AISI and all-cause mortality, and CVD mortality, with adjustment for confounding factors. For the all-cause mortality, age, race, education, marital status, the severity of osteoporosis, PIR, smoking status, total cholesterol, glucose, SBP, DBP, BMI, central obesity, history of CVD, history of fracture, diabetes, lipid-lowering drugs, osteoporosis drugs, occupation, and energy intake were adjusted for; adjusted covariates for the CVD mortality included age, race, education, marital status, the severity of osteoporosis, PIR, HDL-C, total cholesterol, glucose, SBP, DBP, BMI, central obesity, CVD history, fracture history, diabetes, lipid-lowering drugs, and occupation. Subgroup analysis was conducted based on the CVD history (yes or no), history of fracture (yes or no), age ≥ 65 years old (yes or no), BMI > 25 kg/m^2^ (yes or no), if diabetes (yes or no), and if osteoporosis (yes or no). To explore whether our method of handling missing data had an impact on our results, we performed sensitivity analyses.

The area under the curve (AUC) of the receiver operator characteristic curve (ROC) and the concordance index (C-index) were calculated to evaluate the predictive performance of NLR, PLR, MLR, SII, SIRI, AISI in predicting mortality in women with PMOP or osteopenia.

## Results

### Participant selection and baseline characteristics

**Fig. 1:**
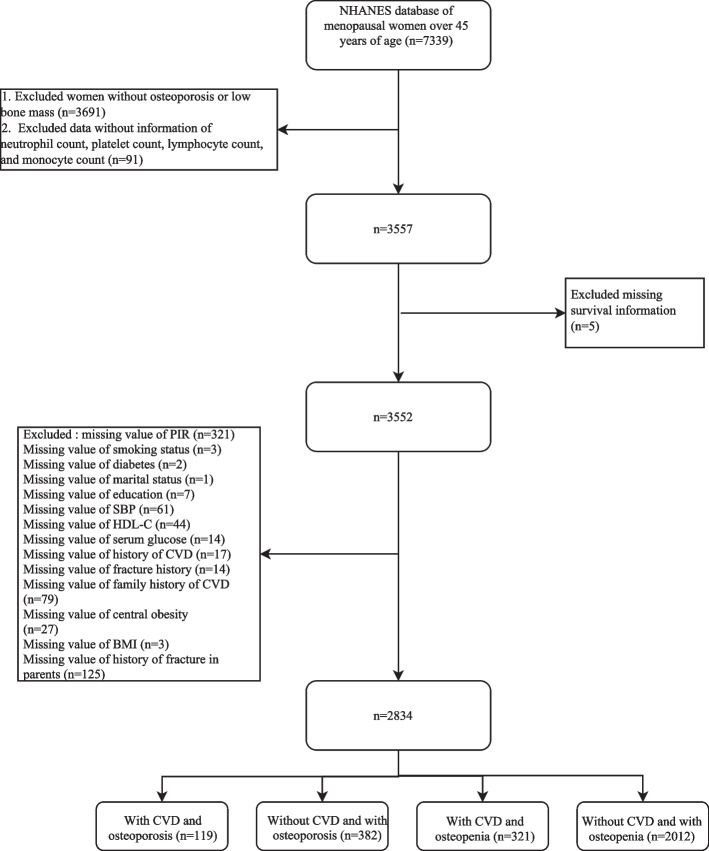
The flow chart of data screening and participant selection. BMI, body mass index; CVD, cardiovascular disease; HDL-C, high-density lipoprotein cholesterol; NHANES, National Health and Nutrition Examination Survey; PIR, poverty income ratio; SBP, systolic blood pressure

Initially, 7,339 postmenopausal women over 45 years were identified from the NHANES database. The flow chart of data screening and participant selection is depicted in Fig. 1. Based on the inclusion and exclusion criteria, 2,834 women were included in this study. The mean age of the women was 64.85 (0.27) years old. The mean survival time was 113.51 (3.15) months. During the follow-up, we found 602 women suffered from all-cause mortality, and 185 women suffered from CVD mortality.

The results showed that there were significant differences in NLR, PLR, MLR, SII, SIRI, AISI, age, race, education, marital status, family income, smoking status, total cholesterol, serum glucose, SBP, DBP, BMI, central obesity, diabetes, history of CVD, energy, lipid-lowering drugs, osteoporosis drugs, the severity of osteoporosis, and survival time between women who suffered from all-cause mortality and those who did not (all *P* < 0.05). The results showed significant differences between women who died of CVD and women who did not die of CVD in terms of NLR, MLR, SIRI, AISI, age, race, education, marital status, family income, HDL-C, total cholesterol, serum glucose, SBP, DBP, central obesity, diabetes and history of CVD, energy, lipid-lowering drugs, osteoporosis drugs (all *P* < 0.05). The characteristics of the study participants are summarized in Table 1.

**Table 1:** Baseline characteristics of included participants

		All-cause mortality			CVD mortality		
Variables	Total (*n* = 2834)	No (*n* = 2232)	Yes (*n* = 602)	*P*	No (*n* = 2,649)	Yes (*n* = 185)	*P*
NLR, Mean (S.E)	2.15 (0.03)	2.09 (0.03)	2.44 (0.06)	< 0.001	2.14 (0.03)	2.38 (0.07)	0.003
PLR, Mean (S.E)	139.48 (1.58)	137.43 (1.88)	148.67 (3.06)	0.003	139.22 (1.70)	143.89 (3.85)	0.297
MLR, Mean (S.E)	0.29 (0.00)	0.28 (0.00)	0.32 (0.01)	< 0.001	0.28 (0.00)	0.33 (0.02)	0.003
SII, Mean (S.E)	555.38 (7.60)	537.15 (8.59)	636.88 (15.10)	< 0.001	552.52 (8.04)	604.40 (24.14)	0.052
SIRI, Mean (S.E)	1.17 (0.02)	1.12 (0.02)	1.43 (0.05)	< 0.001	1.16 (0.02)	1.44 (0.08)	< 0.001
AISI, Mean (S.E)	308.33 (5.29)	292.31 (5.68)	379.95 (12.77)	< 0.001	305.07 (5.40)	364.16 (20.53)	0.007
Age, years, Mean (S.E)	64.85 (0.27)	63.31 (0.30)	71.74 (0.44)	< 0.001	64.36 (0.29)	73.16 (0.93)	< 0.001
Race/ethnicity, n (%)				< 0.001			< 0.001
Mexican American	393 (4.54)	331 (4.77)	62 (3.53)		371 (4.56)	22 (4.18)	
Non-Hispanic White	1672 (81.37)	1245 (80.34)	427 (86.00)		1539 (80.97)	133 (88.30)	
Non-Hispanic Black	310 (4.89)	240 (4.73)	70 (5.61)		290 (4.89)	20 (4.82)	
Other race including multi-racial	459 (9.19)	416 (10.16)	43 (4.86)		449 (9.57)	10 (2.69)	
Education Level, n (%)				< 0.001			0.002
Less than 9th grade	331 (5.54)	251 (4.66)	80 (9.46)		306 (5.31)	25 (9.37)	
9th-11th grade	404 (11.03)	283 (9.60)	121 (17.44)		365 (10.66)	39 (17.41)	
High school grade/GED or equivalent	755 (28.06)	578 (27.37)	177 (31.18)		702 (27.92)	53 (30.50)	
Some college or AA degree	788 (29.05)	643 (29.75)	145 (25.94)		744 (29.28)	44 (25.14)	
College graduate or above	556 (26.31)	477 (28.62)	79 (15.98)		532 (26.82)	24 (17.58)	
Marital status, n (%)				< 0.001			0.020
Married	1360 (54.63)	1147 (58.13)	213 (38.98)		1293 (55.35)	67 (42.27)	
Never married	147 (3.90)	120 (3.85)	27 (4.13)		135 (3.83)	12 (5.18)	
Other (widowed, divorced, separated, living with partner)	1327 (41.47)	965 (38.02)	362 (56.90)		1221 (40.83)	106 (52.55)	
Family PIR, ratio, Mean (S.E)	3.06 (0.05)	3.19 (0.06)	2.51 (0.07)	< 0.001	3.09 (0.06)	2.63 (0.14)	0.003
Smoked at least 100 cigarettes in life,				0.001			0.156
Yes	1130 (42.38)	842 (40.56)	288 (50.51)		1057 (42.68)	73 (37.15)	
No	1704 (57.62)	1390 (59.44)	314 (49.49)		1592 (57.32)	112 (62.85)	
MET, met*min, Mean (S.E)	544.30 (25.20)	565.48 (26.76)	449.65 (64.33)	0.094	547.60 (25.72)	487.97 (83.85)	0.484
Direct HDL-C, mmol/L, Mean (S.E)	1.60 (0.01)	1.61 (0.01)	1.57 (0.03)	0.123	1.61 (0.01)	1.53 (0.04)	0.021
Total cholesterol, mg/dL, Mean (S.E)	210.12 (1.18)	211.44 (1.24)	204.22 (1.78)	< 0.001	210.46 (1.19)	204.15 (3.15)	0.047
Glucose, serum, mmol/L, Mean (S.E)	5.57 (0.04)	5.53 (0.04)	5.75 (0.07)	0.006	5.56 (0.04)	5.85 (0.11)	0.010
SBP, mmHg, Mean (S.E)	129.38 (0.55)	127.88 (0.62)	136.08 (1.11)	< 0.001	128.99 (0.60)	136.06 (1.92)	0.001
DBP, mmHg Mean (S.E)	68.36 (0.45)	69.28 (0.49)	64.28 (0.72)	< 0.001	68.67 (0.46)	63.13 (1.56)	< 0.001
BMI, n (%)				0.003			0.272
Normal\underweight	1074 (41.54)	803 (40.01)	271 (48.38)		988 (41.11)	86 (48.93)	
Overweight	1021 (33.39)	815 (33.88)	206 (31.21)		966 (33.69)	55 (28.40)	
Obese	739 (25.07)	614 (26.11)	125 (20.41)		695 (25.21)	44 (22.66)	
Central obesity, n (%)				0.033			0.035
No	2103 (74.93)	1631 (74.04)	472 (78.89)		1954 (74.50)	149 (82.18)	
Yes	731 (25.07)	601 (25.96)	130 (21.11)		695 (25.50)	36 (17.82)	
Osteoporosis, n (%)				< 0.001			0.002
No	2333 (84.19)	1903 (86.77)	430 (72.67)		2199 (84.69)	134 (75.71)	
Yes	501 (15.81)	329 (13.23)	172 (27.33)		450 (15.31)	51 (24.29)	
Parents ever had fracture, n (%)				0.512			0.728
No	2470 (85.82)	1954 (86.04)	516 (84.84)		2311 (85.88)	159 (84.68)	
Yes	364 (14.18)	278 (13.96)	86 (15.16)		338 (14.12)	26 (15.32)	
Doctor told you have diabetes, n (%)				< 0.001			< 0.001
Yes	406 (10.04)	293 (8.97)	113 (14.84)		363 (9.60)	43 (17.70)	
No	2428 (89.96)	1939 (91.03)	489 (85.16)		2286 (90.40)	142 (82.30)	
Family history of CVD, n (%)				0.401			0.803
Yes	454 (17.26)	355 (16.93)	99 (18.71)		422 (17.21)	32 (18.07)	
No	2380 (82.74)	1877 (83.07)	503 (81.29)		2227 (82.79)	153 (81.93)	
History of CVD, n (%)				< 0.001			< 0.001
Yes	440 (13.01)	271 (9.85)	169 (27.13)		381 (11.97)	59 (30.91)	
No	2394 (86.99)	1961 (90.15)	433 (72.87)		2268 (88.03)	126 (69.09)	
Energy, kcal, Mean (S.E)	1684.80 (19.94)	1710.97 (23.84)	1567.71 (27.62)	< 0.001	1710.97 (23.84)	1567.71 (27.62)	< 0.001
Antihypertensive drug, n (%)				0.094			0.094
No	2506 (89.36)	1987 (89.93)	519 (86.81)		1987 (89.93)	519 (86.81)	
Yes	328 (10.64)	245 (10.07)	83 (13.19)		245 (10.07)	83 (13.19)	
Lipid-lowering drug, n (%)				< 0.001			< 0.001
No	1885 (68.00)	1519 (69.66)	366 (60.55)		1519 (69.66)	366 (60.55)	
Yes	949 (32.00)	713 (30.34)	236 (39.45)		713 (30.34)	236 (39.45)	
Osteoporosis drugs, n (%)				0.002			0.002
No	2545 (89.93)	2028 (90.94)	517 (85.39)		2028 (90.94)	517 (85.39)	
Yes	289 (10.07)	204 (9.06)	85 (14.61)		204 (9.06)	85 (14.61)	
Occupation, n (%)				< 0.001			< 0.001
Employee of private company	632 (28.41)	575 (32.14)	57 (11.71)		575 (32.14)	57 (11.71)	
Work for government	171 (6.68)	160 (7.78)	11 (1.78)		160 (7.78)	11 (1.78)	
Self-employed or family business/farm	119 (4.59)	105 (4.77)	14 (3.79)		105 (4.77)	14 (3.79)	
Unknown	1912 (60.32)	1392 (55.31)	520 (82.71)		1392 (55.31)	520 (82.71)	
Severity of osteoporosis, n (%)				0.003			0.034
Osteoporosis	449 (16.81)	320 (15.60)	129 (22.22)		408 (16.40)	41 (23.78)	
Osteopenia	2385 (83.19)	1912 (84.40)	473 (77.78)		2241 (83.60)	144 (76.22)	
Survival time, month, Mean (S.E)	113.51 (3.15)	117.00 (3.70)	97.92 (3.72)	< 0.001	114.40 (3.41)	98.23 (7.29)	0.064

*AA* Associate of arts, AISI, neutrophil × monocyte × platelet /lymphocyte ratio, *BMI* Body mass index, *CVD* Cardiovascular disease, *DBP* Diastolic blood pressure, *GED* General equivalent diploma, *HDL-C* high density lipoprotein cholesterol, *MET* Metabolic equivalent, *MLR* Monocyte-to-lymphocyte ratio, *NLR* Neutrophil-to-lymphocyte ratio, *PIR* Poverty-to-income ratio, *PLR* Platelet-to-lymphocyte ratio, *SBP* Systolic blood pressure, *SII* Immune-inflammation index, *SIRI* Systemic inflammation response index

Of the 2,834 women, 2,333 had osteopenia, and 501 had osteoporosis. Women with osteoporosis had a higher risk of all-cause mortality (31.61% vs. 15.78%) and CVD mortality (8.48% vs. 4.97%). There were significant differences between women with PMOP and women with postmenopausal osteopenia in NLR, MLR, SII, SIRI, AISI, age, education level, marital status, family PIR, MET, SBP, DBP, BMI, central obesity, history of CVD, history of osteoporosis, osteoporosis medication, occupation, all-cause mortality, CVD mortality, and follow-up time (all *P* < 0.05). Differences in characteristics between women with PMOP and women with postmenopausal osteopenia are shown in Supplementary Table 1.

### The associations of inflammatory markers with all-cause mortality and CVD mortality in postmenopausal women with osteoporosis or osteopenia

From the unadjusted model, we observed that NLR, MLR, SII, SIRI, and AISI were associated with all-cause mortality in postmenopausal women with osteoporosis or osteopenia. Following the adjustments of confounding factors, NLR (HR: 1.17, 95% CI: 1.09 to 1.26, *P* < 0.001), MLR (HR: 1.16, 95%CI: 1.08 to 1.25, *P* < 0.001), SII (HR: 1.11, 95%CI: 1.04 to 1.18, *P* < 0.001), SIRI (HR: 1.21, 95%CI: 1.12 to 1.30, *P* < 0.001), and AISI (HR: 1.13, 95%CI: 1.06 to 1.20, *P* < 0.001) remained significantly associations with all-cause mortality in postmenopausal women with osteoporosis or osteopenia. NLR (HR: 1.16, 95%CI: 1.04 to 1.29, *P* = 0.007), MLR (HR: 1.18, 95%CI: 1.05 to 1.33, *P* = 0.005), SIRI (HR: 1.27, 95%CI: 1.10 to 1.46, *P* < 0.001), and AISI (HR: 1.15, 95%CI: 1.01 to 1.30, *P* = 0.032) were related to CVD mortality in postmenopausal women with osteoporosis or osteopenia. The associations of inflammatory markers with all-cause mortality and CVD mortality in postmenopausal women with osteoporosis or osteopenia are shown in Table 2.

**Table 2:** The associations of inflammatory markers with all-cause mortality and CVD mortality in postmenopausal women with osteoporosis or osteopenia

	All-cause mortality				CVD mortality			
	Unadjusted Model		Adjusted Model		Unadjusted Model		Adjusted Model	
Variables	HR (95%CI)	*P*	HR (95%CI)	*P*	HR (95%CI)	*P*	HR (95%CI)	*P*
NLR	1.24 (1.14–1.34)	< 0.001	1.17 (1.09–1.26)	< 0.001	1.21 (1.11–1.33)	< 0.001	1.16 (1.04–1.29)	0.007
PLR	1.06 (0.97–1.16)	0.224	1.05 (0.98–1.13)	0.131	1.00 (0.86–1.16)	0.972	0.98 (0.88–1.09)	0.669
MLR	1.36 (1.29–1.43)	< 0.001	1.16 (1.08–1.25)	< 0.001	1.41 (1.31–1.52)	< 0.001	1.18 (1.05–1.33)	0.005
SII	1.18 (1.10–1.26)	< 0.001	1.11 (1.04–1.18)	< 0.001	1.12 (1.00–1.26)	0.043	1.09 (0.97–1.23)	0.156
SIRI	1.36 (1.29–1.44)	< 0.001	1.21 (1.12–1.30)	< 0.001	1.37 (1.25–1.51)	< 0.001	1.27 (1.10–1.46)	< 0.001
AISI	1.26 (1.18–1.34)	< 0.001	1.13 (1.06–1.20)	< 0.001	1.24 (1.10–1.39)	< 0.001	1.15 (1.01–1.30)	0.032

AISI, neutrophil × monocyte × platelet /lymphocyte ratio, *CI* Confidence interval, *CVD* Cardiovascular disease, *HR* Hazard ratio, *MLR* Monocyte-to-lymphocyte ratio, *NLR* Neutrophil-to-lymphocyte ratio, *PLR* Platelet-to-lymphocyte ratio, *SII* Immune-inflammation index, *SIRI* Systemic inflammation response index

For the all-cause mortality, age, race, education, marital status, the severity of osteoporosis, poverty-to-income ratio (PIR), smoking status, total cholesterol, glucose, systolic blood pressure (SBP), diastolic blood pressure (DBP), body mass index (BMI), central obesity, history of CVD, history of fracture, diabetes, lipid-lowering drugs, osteoporosis drugs, occupation, and energy intake were adjusted for; adjusted covariates for the CVD mortality included age, race, education, marital status, the severity of osteoporosis, PIR, high density lipoprotein cholesterol (HDL-C), total cholesterol, glucose, SBP, DBP, BMI, central obesity, CVD history, fracture history, diabetes, lipid-lowering drugs, and occupation

**Fig. 2:**
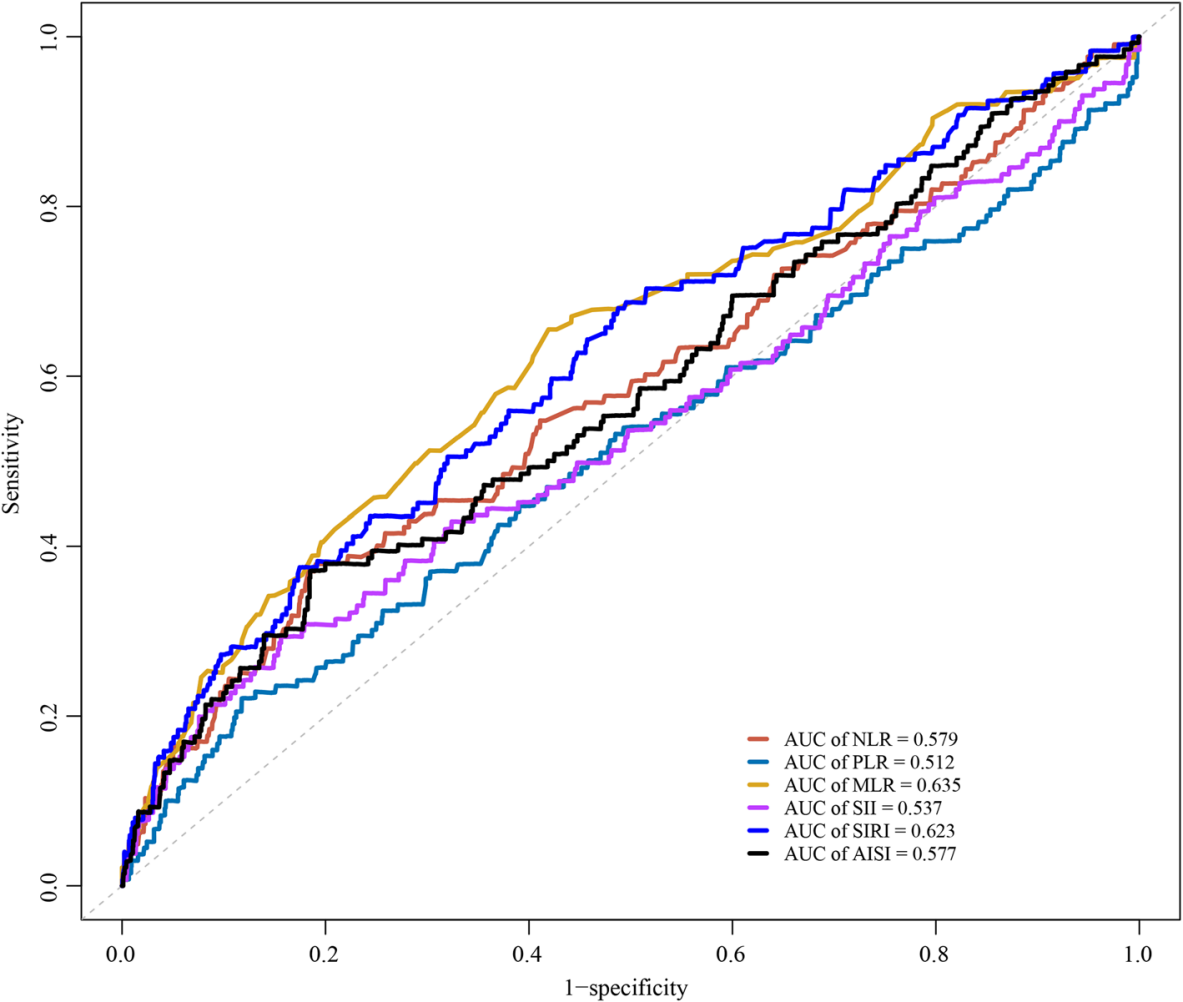
The predictive performances of inflammatory markers for mortality in postmenopausal women with osteoporosis or osteopenia. AISI, neutrophil × monocyte × platelet/lymphocyte ratio; AUC, area under the curve; MLR, monocyte/lymphocyte ratio; NLR, neutrophil/lymphocyte ratio; PLR, platelet/lymphocyte ratio; SII, neutrophil × platelet/lymphocyte; SIRI, neutrophil × monocyte/lymphocyte

The predictive performance of inflammatory markers for all-cause mortality in postmenopausal women with osteoporosis or osteopenia is shown in Fig. 2. Higher AUC values were observed for SIRI (0.63) and MLR (0.635) in predicting all-cause mortality in postmenopausal women with osteoporosis or osteopenia. Table 3 shows the C-index of inflammatory markers for predicting all-cause mortality and CVD mortality in postmenopausal women with osteoporosis or osteopenia. ROCs for comparing performance with and without biomarkers in all-cause and CVD mortality are depicted in supplementary Fig. 1 and Fig. 2.

**Table 3:** The predictive performances of inflammatory markers for all-cause mortality and CVD mortality in postmenopausal women with osteoporosis or osteopenia

	All-cause mortality	CVD mortality
Variables	C-index (95%CI)	C-index
NLR	0.59 (0.56–0.62)	0.59 (0.54–0.64)
PLR	0.51 (0.48–0.54)	0.50 (0.45–0.55)
MLR	0.61 (0.58–0.64)	0.60 (0.55–0.65)
SII	0.55 (0.52–0.59)	0.55 (0.50–0.60)
SIRI	0.62 (0.59–0.64)	0.61 (0.56–0.66)
AISI	0.58 (0.55–0.61)	0.57 (0.52–0.63)

*CVD* Cardiovascular disease, *NLR* Neutrophil-to-lymphocyte ratio, *PLR* Platelet-to-lymphocyte ratio, *MLR* Monocyte-to-lymphocyte ratio, *SII* Immune-inflammation index, *SIRI* Systemic inflammation response index, *AISI* Neutrophil × monocyte × platelet /lymphocyte ratio; confidence interval

### Subgroup analysis of the associations of inflammatory markers with all-cause mortality and CVD mortality in postmenopausal women with osteoporosis or osteopenia

**Fig. 3:**
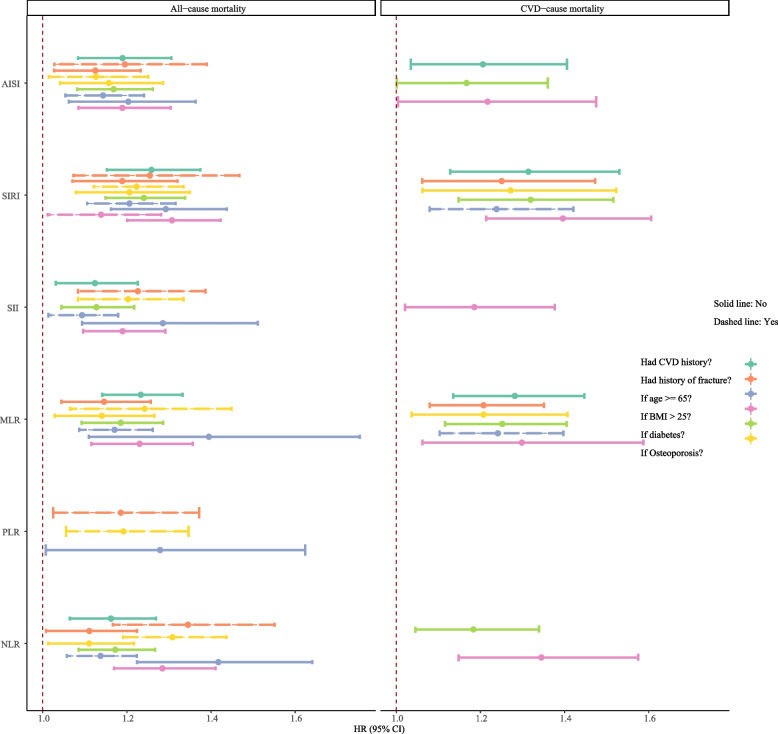
Subgroup analysis of the associations of inflammatory markers with all-cause mortality and cardiovascular mortality in postmenopausal women with osteoporosis or low bone mass. BMI, body mass index; CVD, cardiovascular disease; HR, hazard ratio; CI: confidence interval

Subgroup analysis of the associations of inflammatory markers with all-cause mortality and CVD mortality in postmenopausal women with osteoporosis or osteopenia mass is described in Fig. 3. The subgroup analysis showed that AISI was associated with all-cause mortality and CVD mortality in postmenopausal women who had a CVD history, diabetes, and whose BMI > 25 kg/m^2^. SIRI was associated with all-cause mortality in women who had CVD history and diabetes, and was associated with CVD mortality in postmenopausal women with CVD history, fracture history, osteoporosis, diabetes, and BMI > 25 kg/m^2^. However, SIRI was associated with CVD mortality in postmenopausal women aged < 65 years old. SII was related to all-cause mortality in women with a history of CVD and diabetes, and BMI > 25 kg/m^2^, and without a history of fracture and osteoporosis. Nevertheless, SII was only related to CVD mortality in women with BMI > 25 kg/m^2^. MLR was associated with all-cause mortality among postmenopausal women with CVD history, fracture history, diabetes, and BMI > 25 kg/m^2^, while MLR was associated with CVD mortality in postmenopausal women with CVD history, fracture history, osteoporosis, diabetes, BMI > 25 kg/m^2^, and age < 65 years old. PLR was associated with all-cause mortality among postmenopausal women without a history of fracture and osteoporosis, and aged ≥ 65 years old. NLR was associated with all-cause mortality in postmenopausal women who had CVD and diabetes history, and BMI > 25 kg/m^2^ while only associated with CVD mortality in postmenopausal women who had diabetes history and BMI > 25 kg/m^2^.

## Discussion

Although several drugs have been introduced to menopausal disorder management and bone functions [9, 34, 35], osteoporosis affects postmenopausal women, leading to the deterioration of the microarchitectural bone structure and osteopenia, with an increased risk of fracture and associated disability, morbidity, and mortality [36]. Recently, an increasing number of studies have reported an association between inflammatory biomarkers and fracture outcomes ^[[21, 37]]^. Therefore, this study was conducted to evaluate the association of inflammatory markers with all-cause mortality and CVD mortality in women with PMOP or osteopenia. Our results showed that NLR, MLR, SII, SIRI, and AISI were significantly associated with all-cause mortality in postmenopausal women with osteoporosis or osteopenia. NLR, MLR, SIRI, and AISI were associated with CVD mortality in postmenopausal women with osteoporosis or osteopenia. According to the results of the subgroup analysis, AISI, SIRI, and MLR were associated with all-cause mortality and CVD mortality in postmenopausal women with PMOP or osteopenia who had a history of CVD and diabetes. AISI, SII, MLR, and NLR were associated with all-cause mortality and CVD mortality in women with PMOP or osteopenia and BMI > 25 kg/m^2^. PLR was associated with all-cause mortality in PMOP or osteopenia women aged ≥ 65 years.

Our study revealed that NLR, MLR, SIRI, and AISI were related to all-cause mortality and CVD mortality in postmenopausal women with osteoporosis or osteopenia. An increase in MLR, an inflammatory marker [38], in proportion to inflammation is associated with osteoporosis and bone-derived diseases [14, 39]. The MLR was suggested to be associated with poor overall survival in patients with a variety of cancers [40]. MLR has been found to reflect the severity of systemic inflammation and immune damage [38]. Regarding the NLR, in patients receiving hemodialysis, lower NLR levels are associated with health outcomes [41]. A study [42] by Peng et al. revealed that NLR is a sensitive independent prognostic biomarker in patients with myocardial infarction. Huang et al. [43] found that a high NLR is associated with poor prognosis in osteoporotic individuals. NLR and MLR are measures of acute myeloid-driven innate immune responses reported to chronic, lymphocyte-driven, immunological memory reflected by lymphocyte numbers. An increased MLR and NLR may reflect an immunological imbalance between a potential ongoing clinical or sub-clinical acute inflammation and an impaired immune defense against pathogens [44]. SIRI has recently been proven to reflect the micro-inflammatory state, and it has been proven to be related to the prognosis of many diseases [45–47]. To the best of our knowledge, this is the first study to identify the relationship between SIRI and the risk of mortality in women with PMOP or osteopenia. The underlying mechanism by which SIRI affects the risk of all-cause mortality and CVD mortality in women with PMOP is unclear. SIRI is an indicator of inflammation that integrates three immune pathways including neutrophils and monocytes that account for the persistent inflammatory response, and lymphocytes that account for immune regulation [48, 49]. The higher the ratio, the greater the imbalance, and the more severe the inflammatory response.

In our subgroup analysis, AISI, SII, MLR, and NLR were associated with all-cause mortality and CVD mortality in PMOP or osteopenia women with BMI > 25 kg/m^2^. A rapid increase in BMI related to obesity consequently impairs bone health [50]. The association between inflammatory indicators and morality in obese women with PMOP or osteopenia may be attributed to inflammation. Sub-clinical inflammation accompanying obesity leads to the development of cardiac metabolic complications and insulin resistance [51]. Increasing serum levels of pro-inflammatory cytokines (IL-1, IL-6, and TNF-a) and acute phase proteins in obesity, especially abdominal obesity, are important mediators in bone resorption and osteoclast differentiation [52]. An increase in pro-inflammatory cytokines caused by chronic inflammation results in bone loss and absorption [53]. Our results suggest that inflammatory biomarkers and the risk of mortality among postmenopausal women with osteoporosis and osteopenia may vary according to women’s BMI, but our research at least shows that attention to the inflammatory indicators related to the mortality in obese women may be of great significance to reduce the mortality in obese menopausal women with osteoporosis or osteopenia. According to the results of subgroup analysis, AISI, SIRI, and MLR were associated with all-cause mortality and CVD mortality in postmenopausal women with osteoporosis or osteopenia who had a history of diabetes. The presence of hyperglycemia exacerbates inflammation [54]. PLR was associated with all-cause mortality among PMOP or osteopenia women with age ≥ 65 years old. Dysregulation and overactivation of inflammatory processes in the elderly result in the persistence of chronic inflammatory conditions [55]. All in all, the association between the inflammatory biomarkers and mortality in postmenopausal women with osteoporosis or osteopenia who were older age and had diabetes may attribute to the inflammation. In addition, AISI, SIRI, and MLR were associated with all-cause mortality and CVD mortality in postmenopausal women with osteoporosis or osteopenia who had a CVD history. Given the inconsistent results in different populations in our subgroup analyses, the clinical utility of biomarkers shown here still needs to be determined in PMOP or osteopenia women.

In this study, we also found the predictive values of the inflammatory biomarkers in predicting the mortality in postmenopausal women with osteoporosis or osteopenia, of which higher AUC values were observed for SIRI and MLR in predicting all-cause mortality in postmenopausal women with osteoporosis or osteopenia. According to a previous study, admission MLRs were found to be useful hematological data for the prediction of 30-day and 1-year mortality in geriatric hip fracture patients ^19^. SIRI is a novel marker of microinflammation and has been reported as a predictive factor for the prognosis of several diseases [47, 56]. Use of Cox proportional hazards models, a study [47] by li et al. showed that high SIRI had significant predictive values for all-cause and CVD mortality in patients on peritoneal dialysis. Additionally, the author found SIRI had a prognostic value comparable to the MLR. Based on our findings in this study, SIRI may be a risk stratification indicator for mortality of postmenopausal women with osteoporosis or osteopenia.

Osteoprotegerin (OPG)/receptor activator of nuclear factor-kappaB ligand (RANKL)/RANK system is implicated in both the process of vascular calcification and bone loss [57]. Assays of these factors might help predict CVD and osteoporosis and their associated mortality. However, the biomarkers are considered as a direct, reliable, sensitive, simple, and repeatable method, and could be widely used in clinical practice.

There were inevitably some limitations in our study. Firstly, our study was a retrospective observational study in which confounding factors and selective biases existed. Secondly, age at menopause may also affect our outcomes. Although we were interested in investigating this, we were unable to due to the lack of data available on the information of age at menopause. Thirdly, despite significant findings, the effect sizes of the associations were small. The observed associations are likely to be underestimating or overestimating the true relationship between the biomarkers and mortality in postmenopausal women. These findings, if replicated in clinical studies with more in-depth measures, could suggest a reference for the development of clinical interventions with postmenopausal women. Fourthly, retrospective cohort design of this study did not prove causality. Thus, findings from large and prospective studies may offer an understanding of the association of inflammatory markers with all-cause mortality and CVD mortality in postmenopausal women with PMOP or osteopenia. Finally, even though we calculated the AUC and C-index of related factors to evaluate the predictive performance of the biomarkers, the predictive values were not particularly high. A routine blood examination is easily available in clinical practice and contains abundant information reflecting the systematic inflammation level. The measurement of one or more of these factors as to their ability to predict mortality deserves further study.

Despite these limitations, the study has considerable strengths. This study provides evidence of the association between the biomarkers and mortality risk in postmenopausal women with osteoporosis or osteopenia, offering a significant reference in clinical decision-making. Blood routine examination is easily available in clinical practice and contains abundant information reflecting the systematic inflammation level. Thus, it is attractive to achieve early attention to the association between biomarkers in blood routine examination and mortality in postmenopausal women with PMOP or osteopenia. Moreover, the composite indicator of three and four factors was also calculated that may be more stable and less susceptible to other factors, thus increasing the application value in clinical applications to monitor and assess mortality risk in postmenopausal women with osteoporosis or osteopenia.

## Conclusion

Our findings suggest that NLR, MLR, SII, SIRI, and AISI were significantly associated with all-cause mortality in postmenopausal women with osteoporosis or osteopenia. NLR, MLR, and SIRI were related to CVD mortality in postmenopausal women with osteoporosis or osteopenia. This study may achieve early attention to the association between biomarkers in blood routine examination and prognosis in postmenopausal women with PMOP or osteopenia, providing references for the management of prognosis in postmenopausal women with osteoporosis or osteopenia.

### Supplementary Information

**Additional file 1: Supplementary Figure 1.** ROC for comparing performance with and without biomarkers in all-causemortality.

**Additional file 2: Supplementary Figure 2.** ROC for comparing performance with and without biomarkers in CVD mortality.

**Additional file 3: Supplementary Table 1.** Differences of characteristics between women with postmenopausal osteoporosis and women with postmenopausal osteopenia.

## Data Availability

The datasets generated and/or analyzed during the current study are available in the NHANES database, https://wwwn.cdc.gov/nchs/nhanes/.

## Abbreviations

PMOP: Postmenopausal osteoporosis
BMD: Bone mineral density
CVD: Cardiovascular disease
NLR: Neutrophil/lymphocyte ratio
PLR: Platelet/lymphocyte ratio
MLR: Monocyte/lymphocyte ratio
SII: Systemic immune-inflammation index
SIRI: Systemic inflammation response index
AISI: Neutrophil × monocyte × platelet/lymphocyte ratio
CBC: Complete blood count
NHANES: National Health and Nutrition Examination Survey
DEXA: Dual-energy X-ray absorptiometry
BMI: Body mass index
HDL-C: High-density lipoprotein cholesterol
SBP: Systolic blood pressure
DBP: Diastolic blood pressure
PIR: Poverty income ratio
QA/QC: Quality control and quality assurance protocols
OPG: Osteoprotegerin
RANKL: Receptor activator of nuclear factor-kappaB ligand
