# Supplementary material for: Association of inflammatory markers with all-cause mortality and cardiovascular mortality in postmenopausal women with osteoporosis or osteopenia
Source: BMC Womens Health. 2023 Sep 14;23:487. doi: 10.1186/s12905-023-02631-6 (PMC10500848; doi:10.1186/s12905-023-02631-6)
Supplement: Supplementary file 1 — Additional file 1: Supplementary Figure 1. ROC for comparing performance with and without biomarkers in all-causemortality. [file 12905_2023_2631_MOESM1_ESM.pdf]

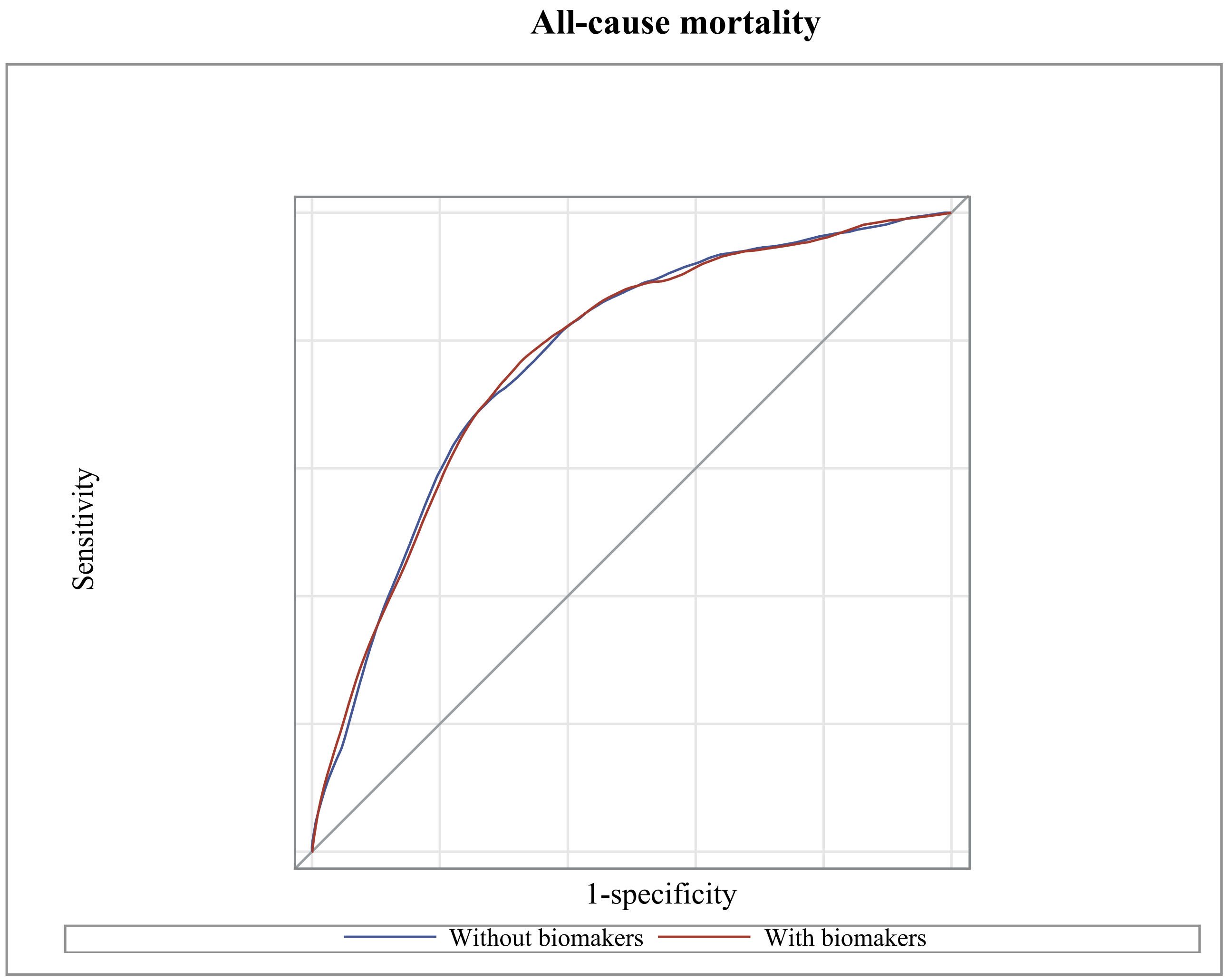

Supplementary Figure 1. ROC for comparing performance with and without biomarkers in all-cause mortality.
