# Supplementary material for: Association of inflammatory markers with all-cause mortality and cardiovascular mortality in postmenopausal women with osteoporosis or osteopenia
Source: BMC Womens Health. 2023 Sep 14;23:487. doi: 10.1186/s12905-023-02631-6 (PMC10500848; doi:10.1186/s12905-023-02631-6)
Supplement: Supplementary file 2 — Additional file 2: Supplementary Figure 2. ROC for comparing performance with and without biomarkers in CVD mortality. [file 12905_2023_2631_MOESM2_ESM.pdf]

CVD-mortality

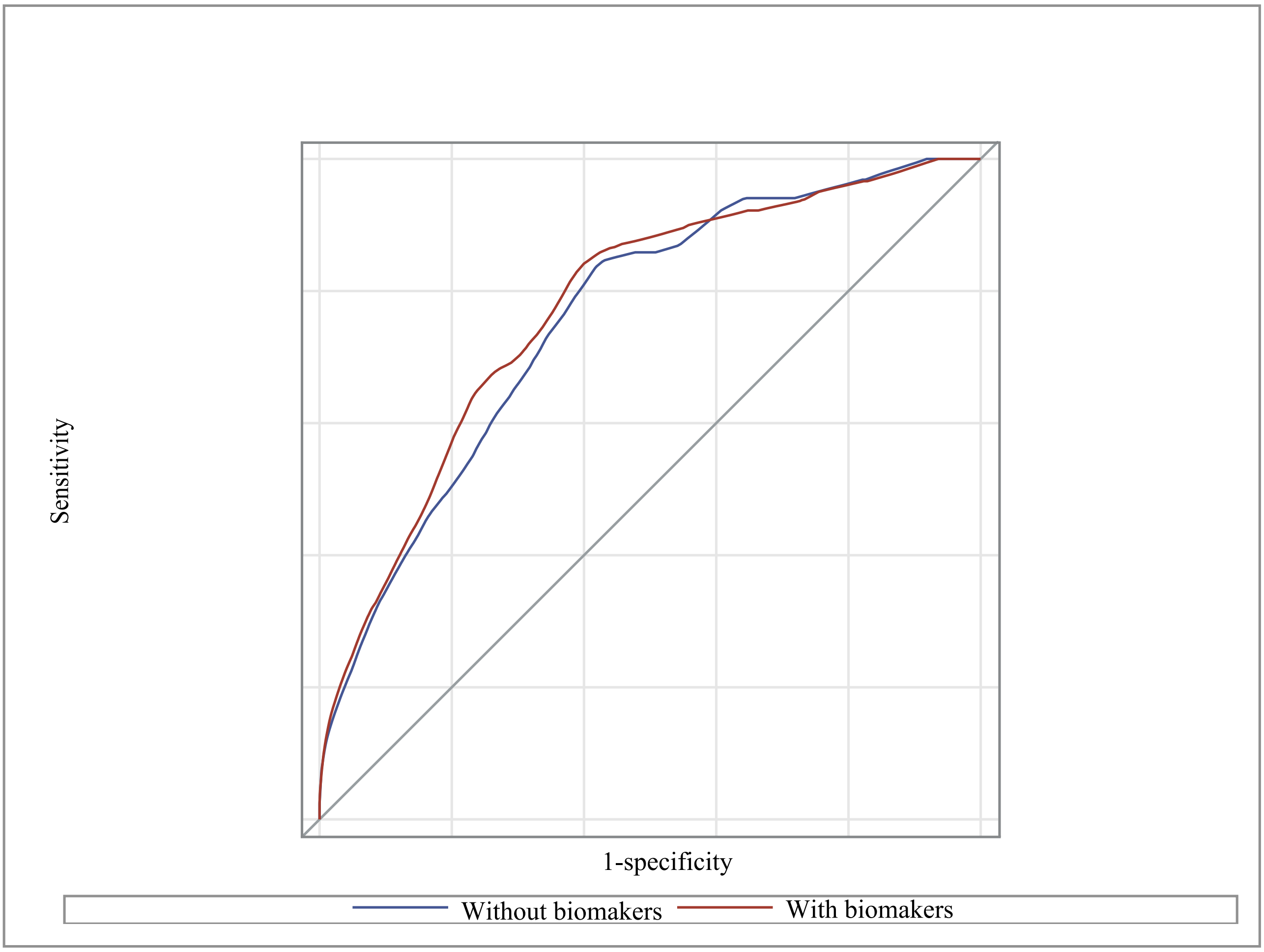

Supplementary Figure 2. ROC for comparing performance with and without biomarkers in CVD mortality.
