# Supplementary material for: Association of inflammatory markers with all-cause mortality and cardiovascular mortality in postmenopausal women with osteoporosis or osteopenia
Source: BMC Womens Health. 2023 Sep 14;23:487. doi: 10.1186/s12905-023-02631-6 (PMC10500848; doi:10.1186/s12905-023-02631-6)
Supplement: Supplementary file 3 — Additional file 3: Supplementary Table 1. Differences of characteristics between women with postmenopausal osteoporosis and women with postmenopausal osteopenia. [file 12905_2023_2631_MOESM3_ESM.rtf]

Supplementary Table 1 Differences of characteristics between women with postmenopausal osteoporosis and women with postmenopausal osteopenia

Variables	Total (n=2834)	Women with osteopenia (n=2333)	Women with osteoporosis (n=501)	statistics	P	
NLR, Mean (S.E)	2.15 (0.03)	2.10 (0.03)	2.46 (0.10)	t=-3.65	<0.001	
PLR, Mean (S.E)	139.48 (1.58)	138.49 (1.77)	144.76 (3.47)	t=-1.59	0.116	
MLR, Mean (S.E)	0.29 (0.00)	0.28 (0.00)	0.31 (0.01)	t=-3.16	0.002	
SII, Mean (S.E)	555.38 (7.60)	538.97 (8.28)	642.80 (23.03)	t=-4.10	<0.001	
SIRI, Mean (S.E)	1.17 (0.02)	1.13 (0.02)	1.43 (0.07)	t=-4.11	<0.001	
AISI, Mean (S.E)	308.33 (5.29)	294.26 (5.64)	383.25 (19.71)	t=-4.12	<0.001	
Age, years, Mean (S.E)	64.85 (0.27)	63.95 (0.27)	69.64 (0.64)	t=-9.17	<0.001	
Race/ethnicity, n (%)				÷2=4.976	0.174	
Mexican American	393 (4.54)	345 (4.71)	48 (3.64)			
Non-Hispanic White	1672 (81.37)	1344 (81.16)	328 (82.52)			
Non-Hispanic Black	310 (4.89)	270 (5.14)	40 (3.54)			
  Other races	459 (9.19)	374 (8.99)	85 (10.30)			
Education Level, n (%)				÷2=21.575	<0.001	
Less than 9th grade	331 (5.54)	251 (4.71)	80 (9.96)			
9-11th grade	404 (11.03)	325 (10.49)	79 (13.91)			
High school grad/GED or equivalent	755 (28.06)	619 (27.96)	136 (28.60)			
Some college or AA degree	788 (29.05)	665 (29.10)	123 (28.80)			
College graduate or above	556 (26.31)	473 (27.74)	83 (18.72)			
Marital status, n (%)				÷2=24.799	<0.001	
  Married	1360 (54.63)	1179 (57.13)	181 (41.30)			
  Never married	147 (3.90)	110 (3.80)	37 (4.44)			
  Others (widowed, divorced, separated, living with partner)	1327 (41.47)	1044 (39.07)	283 (54.27)			
Family PIR, ratio, Mean (S.E)	3.06 (0.05)	3.15 (0.06)	2.61 (0.11)	t=4.50	<0.001	
Smoked at least 100 cigarettes in life,				÷2=2.227	0.136	
Yes	1130 (42.38)	924 (41.60)	206 (46.53)			
No	1704 (57.62)	1409 (58.40)	295 (53.47)			
MET, met*min, Mean (S.E)	544.30 (25.20)	575.21 (27.65)	379.69 (45.39)	t=3.96	<0.001	
Direct HDL-C, mmol/L, Mean (S.E)	1.60 (0.01)	1.61 (0.01)	1.58 (0.03)	t=0.87	0.388	
Total cholesterol, mg/dL, Mean (S.E)	210.12 (1.18)	210.58 (1.22)	207.64 (2.72)	t=1.06	0.292	
Glucose, serum, mmol/L, Mean (S.E)	5.57 (0.04)	5.58 (0.04)	5.53 (0.08)	t=0.65	0.515	
SBP, mmHg, Mean (S.E)	129.38 (0.55)	128.25 (0.53)	135.40 (1.56)	t=-4.60	<0.001	
DBP, mmHg Mean (S.E)	68.36 (0.45)	68.76 (0.47)	66.26 (1.10)	t=2.22	0.030	
BMI, n (%)				÷2=41.392	<0.001	
  Normal\underweight	1074 (41.54)	786 (38.72)	288 (56.58)			
  Overweight	1021 (33.39)	871 (33.83)	150 (31.05)			
  Obese	739 (25.07)	676 (27.45)	63 (12.37)			
Central obesity, n (%)				÷2=10.496	0.001	
  No	2103 (74.93)	1691 (73.52)	412 (82.43)			
  Yes	731 (25.07)	642 (26.48)	89 (17.57)			
Osteoporosis, n (%)				÷2=10.496	0.001	
  No	501 (15.81)	2333 (100.00)				
  Yes			501 (.)			
Parents ever had fracture, n (%)				÷2=0.883	0.347	
  No	2470 (85.82)	2040 (86.17)	430 (83.92)			
  Yes	364 (14.18)	293 (13.83)	71 (16.08)			
Doctor told you have diabetes, n (%)				÷2=0.948	0.330	
  Yes	406 (10.04)	330 (9.80)	76 (11.34)			
  No	2428 (89.96)	2003 (90.20)	425 (88.66)			
Family history of CVD, n (%)				÷2=3.031	0.082	
  Yes	454 (17.26)	366 (16.69)	88 (20.29)			
  No	2380 (82.74)	1967 (83.31)	413 (79.71)			
History of CVD, n (%)				÷2=40.134	<0.001	
  Yes	440 (13.01)	321 (11.17)	119 (22.81)			
  No	2394 (86.99)	2012 (88.83)	382 (77.19)			
History of osteoporosis, n (%)				÷2=22.170	<0.001	
  Yes	449 (16.81)	320 (14.67)	129 (28.20)			
  No	2385 (83.19)	2013 (85.33)	372 (71.80)			
Energy, kcal, Mean (S.E)	1684.80 (19.94)	1692.42 (22.76)	1643.75 (41.21)	t=1.01	0.313	
Antihypertensive drug, n (%)				÷2=1.164	0.281	
  No	2506 (89.36)	2047 (88.99)	459 (91.36)			
  Yes	328 (10.64)	286 (11.01)	42 (8.64)			
Lipid-lowering drug, n (%)				÷2=0.016	0.900	
  No	1885 (68.00)	1552 (67.93)	333 (68.33)			
  Yes	949 (32.00)	781 (32.07)	168 (31.67)			
Osteoporosis drugs, n (%)				÷2=4.986	0.026	
  No	2545 (89.93)	2115 (90.72)	430 (85.71)			
  Yes	289 (10.07)	218 (9.28)	71 (14.29)			
Occupation, n (%)				÷2=67.874	<0.001	
  Employee of private company	632 (28.41)	565 (30.97)	67 (14.75)			
  Work for government	171 (6.68)	156 (7.41)	15 (2.78)			
  Self-employed or family business/farm	119 (4.59)	108 (5.21)	11 (1.29)			
  Unknown	1912 (60.32)	1504 (56.40)	408 (81.18)			
All-cause mortality, n (%)				÷2=47.908	<0.001	
  No	2232 (81.72)	1903 (84.22)	329 (68.39)			
  Yes	602 (18.28)	430 (15.78)	172 (31.61)			
CVD mortality, n (%)				÷2=9.756	0.002	
  No	2649 (94.48)	2199 (95.03)	450 (91.52)			
  Yes	185 (5.52)	134 (4.97)	51 (8.48)			
Follow-up time, month, Mean (S.E)	113.51 (3.15)	115.81 (3.35)	101.25 (4.96)	t=2.88	0.005	
Notes: NLR, neutrophil-to-lymphocyte ratio; PLR, platelet-to-lymphocyte ratio; MLR, monocyte-to-lymphocyte ratio; SII, immune-inflammation index; SIRI, systemic inflammation response index; AISI, neutrophil×monocyte×platelet /lymphocyte ratio; GED, general equivalent diploma; AA: associate of arts; PIR, poverty-to-income ratio; MET, metabolic equivalent; HDL-C, high density lipoprotein cholesterol; SBP, systolic blood pressure; DBP, diastolic blood pressure; BMI, body mass index; CVD, cardiovascular disease.
